# Evaluating early administration of the hydroxymethylglutaryl-CoA reductase inhibitor simvastatin in the prevention and treatment of delirium in critically ill ventilated patients (MoDUS trial): study protocol for a randomized controlled trial

**DOI:** 10.1186/s13063-015-0731-0

**Published:** 2015-05-16

**Authors:** Annalisa Casarin, Daniel F McAuley, Timothy M Alce, Xiaobei Zhao, E Wesley Ely, Jim C Jackson, Cliona McDowell, Ashley Agus, Lynn Murphy, Valerie J Page

**Affiliations:** Intensive Care Unit, Watford General Hospital, West Hertfordshire Hospitals NHS Trust, Vicarage Road, Watford, WD18 0HB UK; Centre for Infection and Immunity, Queen’s University of Belfast, Health Sciences Building, Lisburn Road, Belfast, BT9 7AE UK; Regional Intensive Care Unit, Royal Victoria Hospital, 274 Grosvenor Road, Belfast, BT12 6BA UK; Anaesthetics Department, Neville Hall Hospital, Brecon Road, Abergavenny, NP7 7EG Gwent UK; Vanderbilt University School of Medicine, 2301 Vanderbilt Place, Nashville, TN 37235 USA; Tennessee Valley Veteran’s Affairs Geriatric Research Education and Clinical Center (VA-GRECC), 1310 24th Ave. S, Nashville, TN 37212-2637 USA; Department of Psychiatry, Vanderbilt University Medical Center, 1211 Medical Center Drive, Nashville, TN 37232 USA; Northern Ireland Clinical Trials Unit, Elliott Dynes Building, Royal Hospitals, 274 Grosvenor Road, Belfast, BT12 6BA UK; Faculty of Medicine, Imperial College, South Kensington Campus, London, SW7 2AZ UK

**Keywords:** Delirium prevention, Cognitive impairment, Statins, Beta-amyloid

## Abstract

**Background:**

The incidence of delirium in ventilated patients is estimated at up to 82%, and it is associated with longer intensive care and hospital stays, and long-term cognitive impairment and mortality. The pathophysiology of delirium has been linked with inflammation and neuronal apoptosis. Simvastatin has pleiotropic properties; it penetrates the brain and, as well as reducing cholesterol, reduces inflammation when used at clinically relevant doses over the short term. This is a single centre randomised, controlled trial which aims to test the hypothesis that treatment with simvastatin will modify delirium incidence and outcomes.

**Methods/Design:**

The ongoing study will include 142 adults admitted to the Watford General Hospital Intensive Care Unit who require mechanical ventilation in the first 72 hours of admission. The primary outcome is the number of delirium- and coma-free days in the first 14 days. Secondary outcomes include incidence of delirium, delirium- and coma-free days in the first 28 days, days in delirium and in coma at 14 and 28 days, number of ventilator-free days at 28 days, length of critical care and hospital stay, mortality, cognitive decline and healthcare resource use. Informed consent will be taken from patient’s consultee before randomisation to receive either simvastatin (80 mg) or placebo once daily. Daily data will be recorded until day 28 after randomisation or until discharge from the ICU if sooner. Surviving patients will be followed up on at six months from discharge. Plasma and urine samples will be taken to investigate the biological effect of simvastatin on systemic markers of inflammation, as related to the number of delirium- and coma-free days, and the potential of cholinesterase activity and beta-amyloid as predictors of the risk of delirium and long-term cognitive impairment.

**Discussion:**

This trial will test the efficacy of simvastatin on reducing delirium in the critically ill. If patients receiving the statin show a reduced number of days in delirium compared with the placebo group, the inflammatory theory implicated in the pathogenesis of delirium will be strengthened.

**Trial registration:**

The trial was registered with the International Standard Randomised Controlled Trial Registry (ISRCTN89079989) on 26 March 2013.

## Background

Delirium in critically ill patients is a manifestation of acute brain dysfunction. The incidence of delirium in mechanically ventilated patients, assessed using the Confusion Assessment Method for the Intensive Care Unit (CAM-ICU), is estimated at up to 82% [1]. Delirium and dementia are typically presented as separate entities, distinguished by the timing and duration of symptoms, but increasingly are thought to share common pathological mechanisms [2,3], Although the mechanisms by which delirium may predispose patients to long-term cognitive impairment after critical illness have not yet been elucidated, delirium is associated with inflammation and neuronal apoptosis, which may lead to brain atrophy [4]. A longer duration of delirium has been shown to be associated with worse global cognition at three and 12 months after ICU discharge, and with significantly higher ICU and hospital costs [5,6]. An intervention which reduces delirium could potentially translate to a reduction of long-term cognitive impairment and dementia [7].

There is little evidence that commonly used pharmacologic approaches to prevent or treat delirium during critical illness are effective [8,9]. Statins, in addition to decreasing cholesterol synthesis, have complex pleiotropic effects and have been hypothesised to both prevent and treat delirium via effects that may modulate the molecular pathways of inflammation and microglial activation [10].

An *in vitro* study comparing different statins for prevention of neurodegenerative conditions concluded that monacolin J derivatives (natural and semi-synthetic statins), which includes simvastatin, are the best candidates. This is due to their better lipophilicity and capacity to penetrate the blood–brain barrier, cholesterol-lowering effect on neurons with a satisfactory safety profile and *in vitro* protection against neuronal cell death caused by okadaic acid [11]. The aim of the Modifying Delirium Using Simvastatin (MoDUS) trial is to test the hypothesis that treatment with enteral or oral simvastatin (80 mg) will increase the number of delirium- and coma-free days in ventilated ICU patients up to 14 days. The effects of simvastatin treatment on biological mechanisms important in the development of delirium will also be explored, including inflammation biomarkers, anticholinergic activity and beta-amyloid (β-amyloid) [12,13].

### Methods/Design

West Hertfordshire Hospitals National Health Service (NHS) Trust is the Sponsor for the MoDUS trial. The trial will be conducted in accordance with the ethical principles that have their origin in the Declaration of Helsinki. The protocol is approved by the Newcastle and North Tyneside 1 Research Ethics Committee (REC reference: 12/NE/0383, 6 December 2012) and by the Hertfordshire Hospitals Research and Development consortium at Watford General Hospital. The trial is registered on the International Standard Randomised Controlled Trial Registry (ISRCTN89079989, assigned on 26 March 2013). It is funded by the Research for Patients Benefit programme, which is funded and managed by the National Institute for Health Research (NIHR). The trial is being coordinated by West Hertfordshire Hospitals NHS Trust, in partnership with Northern Ireland Clinical Trials Unit (NICTU). The trial will comply with the principles of Good Clinical Practice (GCP) and will be carried out in accordance with applicable legislation and the Standard Operating Procedures of West Hertfordshire Hospitals NHS Trust and the NICTU. The trial will be reported in line with the Consolidated Standards of Reporting Trials (CONSORT) 2010 guidelines [14].

The MoDUS trial is a single centre, randomised, double-blind, placebo-controlled, superiority phase II trial including adults admitted to the Watford General Hospital ICU who require mechanical ventilation in the first 72 hours of admission. The study has three distinct objectives: to investigate the efficacy of early statins administration during an ICU stay for the prevention of ICU delirium; to determine any improvement in related neurocognitive sequelae coupled with standard clinical outcomes; and to study the biological effect of simvastatin on systemic markers of inflammation as related to the number of delirium- and coma-free days, and the potential of cholinesterase activity and β-amyloid as predictors of the risk of delirium and long-term cognitive impairment [12,13].

### Eligibility criteria

Patients will be eligible for the trial if they fulfil the following criterion: adult patients in intensive care requiring intubation within the first 72 hours of admission. Patients fulfilling any of the criteria below will be excluded from the trial:

1. Aged less than 18-years-old;
2. Patient known to be pregnant or breastfeeding;
3. Known allergy to statin drugs;
4. Creatine kinase (CK) level more than 10 times upper limit of normal range within 72 hours of randomisation;
5. Alanine transaminase (ALT) level more than eight times the upper limit of normal range within 72 hours of randomisation;
6. Patients currently receiving ongoing and sustained treatment with any of the following; itraconazole, ketoconazole, HIV protease inhibitors, nefazodone, cyclosporine, amiodarone, verapamil, diltiazem, gemfibrozil or danazol;
7. Uncomplicated elective surgery (planned admission, surgical procedure and recovery as predicted);
8. Patient expected to be discharged within 48 hours of admission;
9. Patients with severe renal impairment (estimated creatinine clearance less than 30 ml/minute) not receiving renal replacement therapy;
10. Severe liver disease (Childs-Pugh score >12);
11. Current or recent treatment (within two weeks) with statins, as statins will be continued assuming there are no contraindications according to normal unit practice;
12. Physician decision that a statin is required for proven indication;
13. Contraindication to enteral drug administration, such as patients with mechanical bowel obstruction (patients with high gastric aspirates due to an ileus are not excluded);
14. Known participation in investigational medicinal product trials within previous 30 days;
15. Consent declined;
16. Treatment withdrawal likely within 48 hours;
17. Non-English speaking patients or those who do not adequately understand verbal or written information or
18. History of porphyria.

### Clinical outcome measures

The primary outcome of the MoDUS trial will be the number of delirium- and coma-free days in the first 14 days after randomization, during which the patient is alive and free from delirium and coma, and where days are counted as calendar days (from 00:00 to 23:59). The coma state in an ICU patient is determined by nurses using the Richmond Agitation and Sedation Score (RASS) and delirium is screened for using the CAM-ICU [15,16]. Patients are defined as delirious if they are awake enough to at least respond to verbal stimulation with eye opening and eye contact for less than 10 seconds (RASS score of −2) and screen positive for delirium. CAM-ICU needs to be negative at all assessments throughout a whole study day for the patient to be considered free from delirium.

There are a number of secondary outcomes in this trial, including: clinical outcomes, biological mechanisms, safety measures and outcomes related to the health economic evaluation. The clinical outcomes are incidence of delirium, delirium- and coma-free days in the first 28 days, delirium at 14 and 28 days, and days in coma at 14 and 28 days. For this investigation, a RASS score of −3, (movement or eye opening in response to voice but no eye contact) regardless of whether disease or sedative induced, is handled as part of the spectrum of coma. While the validation studies for the CAM-ICU have demonstrated that delirium can be diagnosed in patients with a RASS score of −3 at Watford, along with some other centres, we routinely classify a patient with such as score as unable to be assessed (usually due to sedation). We decided this because of the clinical relevance of a RASS score of −3 as shown by the Sedation Practice in Intensive Care Evaluation (SPICE) Study Investigators and ANZICS Clinical Trials Group who have demonstrated that early deep sedation, defined as RASS score −3 to −5, predicts delayed extubation and increased mortality [17].

Other secondary outcomes are the number of ventilator-free days at 28 days, mortality at six months, organ failure-free days, cognitive decline and healthcare resource use. Cognitive impairment at six months is assessed using the telephone interview of cognitive status, called Brief Test of Adult Cognition by Telephone (BTACT), which is a battery of cognitive tests validated for telephone use assessing immediate and delayed memory recall, working memory span, verbal fluency, inductive reasoning and speed of processing [18]. Cognitive decline will be estimated by taking into account the patient’s baseline cognitive function before admission to the ICU, as assessed using the Informant Questionnaire on Cognitive Decline in the Elderly (IQCODE), scored at recruitment and six months later [19].

### Biological mechanisms

A translational study of biological markers of inflammation, cholinergic activity and neurodegeneration to provide insight into the pathophysiology of delirium and the potential of β-amyloid as a predictor of the risk of lon- term cognitive impairment will be undertaken. Blood and urine are taken at baseline prior to study drug administration (day one), and on day three, seven, 14 and 28 (within 6 hours of study drug adminstration) while the patients continue to receive the study drug. Plasma inflammatory response biomarkers, which will include serum C-reactive protein (CRP, cytokines (including tumour necrosis factor alpha(TNF-α, interleukin (IL)-1, IL-6 and IL-8), proteases and anti-proteases, adhesion molecule expression, and coagulation factors will be analysed. In addition simvastatin plasma levels will be analysed.

Plasma β-amyloid measurement has emerged as a promising biomarker to identify elderly persons at risk of developing dementia [20]. Lower β-amyloid 42 and 42/40 levels have been associated with increased risk of developing Alzheimer’s disease. A study suggests that older adults without dementia and with lower β-amyloid 42/40 levels have an increased rate of cognitive decline over nine years compared with those with higher levels [21]. It may be that plasma β-amyloid 42/40 levels may be a predictor of those patients who are most at risk of developing cognitive impairment following ICU delirium, therefore serial β-amyloid 42 and 42/40 ratio levels will be measured. Delirium is associated not only with an unbalanced inflammatory response, but also with a dysfunctional interaction between the cholinergic and immune systems. In order to increase the understanding of the relationship between the cholinergic and immune systems with regard to delirium pathophysiology, we will also measure biomarkers plasma acetylcholinesterase and butyrylcholinesterase, as indicators of cholinergic function [22].

### Safety

Plasma will be collected from each patient on days three, seven, 14, 21 and 28 while the patient is on the ICU, for the purposes of safety monitoring. CK and ALT levels will be measured. The frequency of the following events will be reported: CK level over 10 times the upper limit of normal; ALT level over eight times the upper limit of normal; patients whose CK is elevated more than 10-fold who then require renal replacement therapy; serious adverse events (SAEs) and occurrence of suspected unexpected serious adverse reactions (SUSARs). If the patient needs an acute administration of amiodarone (manufacturer: Ibigen, Aprilia, Italy), the regime of the study drug will be changed: the patient will receive one tablet every other day.

### Health economic evaluation

Health-related quality of life will be measured using the EuroQol 5-dimension 5-level (EQ-5D-5 L) questionnaire [23] at the point of consent to continue and at six months post-randomisation, to calculate quality-adjusted life years (QALY) at six months. Data on the health and social care services used by all patients will be collected for the six-month post-randomisation period. Length of ICU stay (level three care), length of high dependency unit stay (level two care) and length of hospital stay will be recorded in the case report form (CRF), and patients will use logs to record post-hospital discharge health service use. They will be asked to refer to this when completing a questionnaire at six months. If questionnaires are not returned, non-responders will be contacted by telephone and a second questionnaire will be sent out if required. The cost-effectiveness of the treatment compared to the placebo will be assessed.

### Power and sample size estimate

The sample size calculation for this study has been initially based on data from the Awakening and Breathing Controlled trial published in 2008, using the mean delirium- and coma-free days of 10 at 28 days among patients without the study intervention [24]. Our pilot data showed a standard deviation of 4.14 in delirium and coma free days at day 14 in a similar cohort (Shintani A, personal communication, September 2012) Assuming this standard deviation, and assuming a type I error of 0.05 and 80% power, a sample size of 64 patients per group is adequately powered to detect a difference of approximately 2 DCFDs between the intervention and control groups, or approximately 0.5 SD. Allowing for an estimated 10% loss to follow-up, the sample size required is 142. nQuery AdvisoR version 4.0 was used for the sample size analysis (Elashoff, JD 2000).

**Figure 1:**
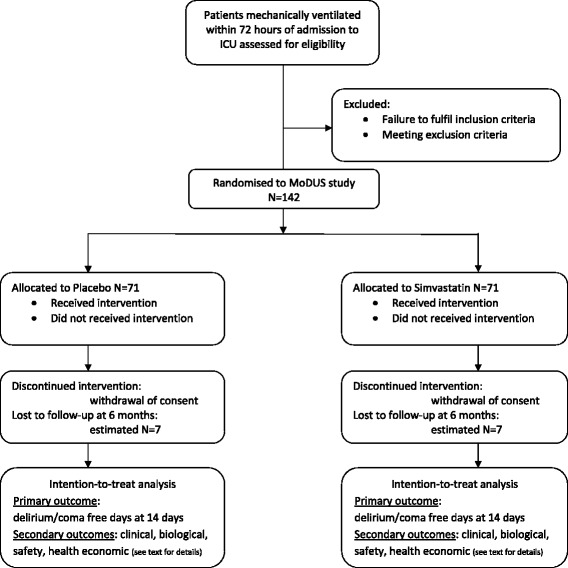
Flow diagram (following CONSORT 2010 Statement Guidelines).

A flow chart for the protocol is outlined in Figure 1.

### Trial conduct

The study will be conducted in accordance with the ethical principles that have their origin in the Declaration of Helsinki. Critically ill patients requiring mechanical ventilation within 72 hours of admission to intensive care will be assessed for eligibility. A screening log will be retained and anonymized. Written informed consent or assent will be gained for patients fulfilling inclusion criteria before beginning any research activities, which include taking baseline bloods. Consent will be sought from the patients themselves if this is possible, but it is recognised that in the majority of cases ICU patients will be unable to give informed consent due to delirium itself, or alterations in their level of consciousness caused by illness and therapeutic sedation. In this situation, consent will be sought from a personal legal representative (PerLR), who may be a relative, partner or close friend, or a professional legal representative (ProfLR), such as a doctor who is not connected with the conduct of the trial. The consent from the representative will remain valid until a decision on consent to continue is obtained from the patient. All surviving patients that regain capacity will be informed about the trial at the earliest opportunity, and asked for consent to continue the study. Patients may withdraw from the trial or be withdrawn by their PerLR or ProfLR at any time, without prejudice. Data recorded up to the point of withdrawal will be included in the trial analysis. The IQCODE for each patient will be completed by the PerLR, any close family members or a friend at the time of consent and recruitment. The patient registration form will be completed and submitted to the NICTU to confirm registration of the patient to the trial, in accordance with the study drug randomisation schedule, and the unique trial identifying number will be assigned at the time of recruitment.

Patient drug packs will be prepared by Victoria Pharmaceuticals (Belfast, UK), and distributed to the site. The 142 consecutive packs will contain either simvastatin or placebo according to a prearranged order dictated by the 1:1 variable block randomisation schedule decided by the use of a random number generator. Simvastatin (40 mg tablets) or identical placebo tablets will be packaged in a white opaque high-density polyethylene plastic container, which will be sealed with a tamper-evident seal and labelled in compliance with applicable regulatory requirements. The placebo and active tablets will be indistinguishable when crushed and dispersed in water for nasogastric tube administration. Each container will contain 70 tablets of the study drug for the treatment of one patient for 28 days (plus seven days coverage). Each pack will be numbered with the patient’s trial identifier. Treatment allocation will be blinded as all trial drugs will be packaged identically and identified only by a unique trial identifier. Recruited patients will be randomised to receive once daily simvastatin (80 mg, as two 40 mg tablets) or two identical placebo tablets administered enterally via a feeding tube or orally for up to 28 days.

Although the primary outcome is determined at 14 days, the study drug will be administered for up to 28 days in order explore if longer treatment is associated with sustained and greater effect; however, the 28 day measurement will be less reliable given an expected median ICU length of stay of nine days. The recruiting clinician will complete a trial prescription form detailing the unique trial identifier assigned to the patient at the time of randomisation, which also corresponds to the study drug pack to be prescribed. The first dose of the study drug will be administered as soon as possible, ideally within four hours of randomisation and after baseline samples are collected, and subsequent doses will be given at 12:00 am on the following calendar day. If, for any reason, a dose is not administered at the intended time, it may be administered subsequently, but not more than 12 hours after the intended time of administration. Any missed doses will be recorded in the CRF to monitor treatment compliance.

Delirium assessment using CAM-ICU will be performed twice by the bedside nurse per nursing shift, with a minimum of four hours between assessments. CAM-ICU is a routine assessment in all ICU patients, and nurses undergo regular updates and have regular access to advice from the ICU delirium study team. Blood and urine samples will be taken at baseline prior to study drug administration (day one) and on day three, seven, 14 and 28 (within 6 hours of study drug adminstration), while patients will continue to receive the study drug for analysis of inflammatory cytokines, β-amyloid and cholinesterase activity. The study drug will be discontinued if any one of the following conditions is met, prior to the maximum treatment period (28 days from randomisation):

1. Study drug-related adverse event, defined as:
  - CK level over 10 times the upper limit of normal;
  - ALT level over eight times the upper limit of normal.
2. Development of a clinical condition requiring immediate treatment with a statin;
3. Discharge from the critical care environment;
4. Death;
5. Discontinuation of active medical treatment;
6. Patient or relative request that the study drug should be discontinued or
7. Decision by the attending clinician that the study drug should be discontinued on safety grounds.

At consent to continue, surviving patients who consented to the follow-up protocol will be approached and EQ-5D-5 L will be completed face-to-face. They will receive a service use log to record their use of health and social care services from hospital discharge until six months post-randomisation. All survivors will be contacted at six months by phone for an assessment of cognitive function using the BTACT. Any deaths after discharge from hospital will be identified using the NHS Patient Centre Database, in order to avoid contacting relatives of patients who have died. Trial patients will be asked to let the Chief Investigator (CI) know if they move house at any time after hospital discharge; the NHS Patient Centre Database will enable us to locate any who move without informing the CI. Patients will be posted a follow-up package at six months post-randomisation, which will include the IQCODE, the EQ-5D-5 L and the use of health and social care services questionnaire.

### Clinical management of patients in the trial

Patients involved in this trial will be managed according to best practice established on the ICU. In patients where there is a clinical indication for acute and immediate treatment with a statin after randomisation, such as an acute myocardial infarction, the study drug will be discontinued and a statin commenced. The patient will not be unblinded and data collection will continue. This will be recorded on the CRF. The number and dose of any antipsychotics or additional psychotropic drugs given to a study patient in the first 28 days or before discharge from the ICU (whichever is the earlier) will be recorded in the CRF. The daily sedation goal for all patients will be a RASS score of 0 to −1, unless a patient’s clinical condition requires a deeper level of sedation, such as a patient with severe acute respiratory distress syndrome, or the patient is weaned from ventilation. The sedative drugs used before patients are consented for the study will be decided by the responsible intensivist. Once they have consented to participate in the study, patients will be maintained using fentanyl (manufacturer: Hameln Pharmaceuticals, Gloucester, UK) and propofol (manufacturer: UAB Norameda, Klaipeda, Lithuania) infusions as needed while requiring ventilation. The infusion rate will be titrated according to RASS assessment and the nurse’s judgment of when a patient is experiencing pain. Daily sedation breaks and daily spontaneous breathing trials will be done at the discretion of the consultant intensivist responsible for clinical management, according to routine unit practice. The sedative infusions will not be restarted as long as the patient tolerates the interruption of sedation drugs.

### Study procedures for unblinding

As a placebo-controlled, double-blind trial, patients and clinicians will be blinded to each patient’s allocation. All trial drugs, whether simvastatin or placebo, will be packaged identically and identified only by a unique trial identifier. Any consultant intensivist may request emergency unblinding on safety grounds. This option should be used only if the patient’s future treatment requires knowledge of the treatment assignment. If a consultant decides that there is justification to unblind a patient, they should attempt to contact the CI or site co-investigator, who will arrange for them to discuss the necessity of unblinding with a clinical member of the trial team. Emergency unblinding will be performed by the on-call pharmacist. The NICTU should also be informed that unblinding has occurred. All events will be logged.

### Data collection and management

All study data for an individual patient will be recorded in the CRF. Patients are identified by initials and their unique trial identifier, which was allocated at the time of randomisation. Data will be collected from the time the patients are enrolled into the trial through to and following their discharge from hospital, up to a maximum of six months following recruitment. Any history of alcohol abuse will be recorded if documented in the medical records. PREdiction of DELIRium in ICu patients (PRE-DELIRIC) and daily Sequential Organ Failure Assessment (SOFA) scores will be calculated using data from patients’ clinical notes. The PRE-DELIRIC is a validated delirium prediction tool for adult intensive care patients [25]. The SOFA score will be used as daily severity of illness assessment of ICU patients.

All completed CRFs will be forwarded to the NICTU, and submitted data will be reviewed and processed as per the NICTU Standard Operating Procedures. Due care will be taken to ensure data safety and integrity, and compliance with the Data Protection Act 1998. The trial identifier, name, address and other contact details of all patients who survive will be kept separately to allow patients to be contacted at six months for the telephone cognitive status evaluation, and for the follow-up questionnaires to be posted to them. No patient-identifiable data will be submitted to the NICTU. All essential documentation and trial records will be stored in conformance with the applicable regulatory requirements, and access to stored information will be restricted to authorised personnel. Trial documentation and data will be archived for at least five years after completion of the trial and as per sponsor requirements.

### Pharmacovigilance

EU Clinical Trials Directive 2001/20 provides the definitions of adverse events and reactions used in this trial (Table 1).

**Table 1:** **Definition of adverse events and reactions according to EU clinical trial directive 2001/20**

**Term**	**Definition**
Adverse Event (AE)	Any untoward medical occurrence in a patient or clinical trial subject to whom a medicinal product has been administered, including occurrences which are not necessarily caused by or related to that product.
Adverse Reaction (AR)	Any untoward and unintended response to an investigational medicinal product related to any dose administered.
Unexpected Adverse Reaction (UAR)	An adverse reaction, the nature or severity of which is not consistent with the information about the medicinal product in question set out in the summary of product characteristics (or Investigator brochure) for that product.
Serious Adverse Event (SAE), Serious Adverse Reaction (SAR) or Suspected Unexpected Serious Adverse Reaction (SUSAR)	Respectively, any adverse event, adverse reaction or unexpected adverse reaction that:
	● results in death
	● is life-threatening
	● requires hospitalization or prolongation of existing hospitalization
	● results in persistent or significant disability or incapacity
	● consists of a congenital anomaly or birth defect^1^
	● is any other important medical event(s) that carries a real, not hypothetical, risk of one of the outcomes above

^1^For this trial it is anticipated that all women of child bearing age admitted will have a pregnancy test. If, however, a subsequent pregnancy is discovered the pregnancy will be followed in order to assess the outcome regarding any adverse event.

Because this study is recruiting a population that is already in a life-threatening condition, it is expected that many of the participants will experience adverse events. Events that are expected in this population (that is, events that are in keeping with the patient’s underlying medical condition) will not be reported as adverse events. An adverse reaction is an adverse event which is related to the administration of the study drug. If any adverse reactions arise, they will be reported on the adverse event form within the CRF. If an SAE thought to be related with the study drug occurs, reporting will follow the regulatory requirements and will be reported to the NICTU and the sponsor within 24 hours of becoming aware of the event. All SUSARs will be the subject of expedited reporting.

### End of trial

The trial will end when 142 patients have been recruited. The Data Monitoring and Ethics Committee (DMEC) will meet every nine months to determine whether there is any reason why recruitment should not continue. Interim analyses will only be undertaken at the request of the DMEC and will only be available to the DMEC. The trial will be stopped prematurely if mandated by the Ethics Committee, mandated by the Medicines and Healthcare products Regulatory Agency, decided by the Trial Steering Committee (TSC) or Sponsor after consideration of recommendations from the DMEC or funding for the trial ceases. The main criteria for early stopping of the trial by the TSC upon suggestions from the DMEC will be that evidence provided by the trial investigators from the trial and from other sources suggests: proof beyond all reasonable doubt that for all, or for some types of patients, the treatment regimen is clearly indicated or contraindicated, and evidence that might reasonably be expected to influence routine clinical practice.

### Statistical analysis plan

Analyses of effectiveness endpoints will be on an intention-to-treat basis. The primary outcome (delirium/coma free days) is a heavily skewed distribution (bimodal with peaks at 0 and 14 days) and the groups will initially be analyzed by t-test with difference in means and 95% confidence intervals (CI) presented. A secondary analysis of these outcome measures involving a bootstrapped t-test will also be conducted to support the findings of the t-tests and confirmed using the Mann–Whitney non-parametric test. Dichotomous outcomes will be analysed using risk ratios and 95% confidence intervals, where appropriate. Continuous variables over time will be analysed by generalised linear modelling. Time to event outcomes such as duration of hospital stay will be analysed by survival methods and reported as hazard ratios and 95% CIs. Primary analyses will be based on patients with outcome data (that is, available case analysis). The randomisation process will ensure balance in baseline variables. A detailed analysis plan will be developed during the trial and submitted to the DMEC for review prior to the commencement of analysis. All analyses will be conducted at the 5% level of significance unless adjustment for multiple testing is needed.

### Health economic analysis

A within-trial cost-utility analysis (CUA) will be undertaken by the health economist to assess the cost-effectiveness of simvastatin compared with placebo in the prevention and treatment of delirium. The perspective of the analysis will be the NHS and Personal Social Services. The outcome of the CUA will be the cost per QALY gained. Patients’ EQ-5D-5 L responses will be presented as utility scores and used to calculate QALYs at six months. Resource use will be valued using national sources of unit costs, such as the NHS Reference Costs and Personal Social Services Research Unit Costs of Health and Social Care [26,27]. Multiple regression models will be used to estimate mean costs and QALYs for the six-month study period, adjusted for covariates. An incremental cost-effectiveness ratio will be calculated to estimate the cost per QALY gained. Patient-level cost and QALY data will be bootstrapped, and a cost-effectiveness acceptability curve will be derived to show the probability of simvastatin being cost-effective compared to a placebo, at various thresholds of willingness-to-pay for an additional QALY. Sensitivity analysis will be performed to explore the impact on cost-effectiveness of variations in key parameters. Given the study timeframe, discounting will not be necessary.

### Trial oversight

The CI will assume overall responsibility for ensuring the trial is run in accordance with GCP and to the highest ethical and scientific standards. West Hertfordshire Hospitals NHS Trust Research and Development Department will be responsible for overseeing the financial management of the trial, and as Sponsors, for ensuring that statutory regulations and reporting requirements are fulfilled. Day-to-day management of the trial will be undertaken by the local study team who will liaise on monthly basis with the Trials Management Group, composed of the CI and supporting staff both at the site and at the NICTU.

The TSC will be comprised of experienced critical care personnel and trialists, as well as a lay representative from ICUsteps, an ICU patient support charity. This charitable trust was founded in 2005 by ex-patients, their relatives and ICU staff to support patients and their families through the recovery from critical illness. The TSC will take responsibility for major decisions, such as a need to change the protocol for any reason, monitoring and supervising the progress of the trial, reviewing relevant information from other sources, considering recommendations from the DMEC, and informing and advising on all aspects of the trial. The DMEC (equivalent to a North American Data Safety Monitoring Board) will be appointed independently from the study team, comprising of at least two clinicians with experience in undertaking clinical trials and caring for critically ill patients and a statistician. The study statistician will provide a report to the DMEC. The DMEC will function primarily as a check for safety reviewing adverse events. They will specifically review the incidence and severity of side effects, SAEs and SUSARs, and produce a safety recommendation following each meeting.

On-site monitoring visits will be undertaken by the Clinical Trials Unit, and will be conducted in accordance with the study monitoring plan. On-site monitoring will be an ongoing activity from the time of initiation until study close-out, and will comply with the principles of GCP and EU directive 2001/20/EC.

Before the study starts, an initiation visit will take place to ensure that all relevant essential documents and trial supplies are in place, and that site staff are fully aware of the study protocol and Standard Operating Procedures. During the study, on-site monitoring visits will check the following: the completeness of patient records; the accuracy of entries on CRFs; the adherence to the protocol, Standard Operating Procedures and GCP and the progress of patient recruitment. Monitoring will also ensure that the study drug is being stored, dispensed and accounted for according to specifications.

## Discussion

There is emerging evidence that statins may have a beneficial role in the prevention and management of delirium in the critically ill. A recent observational study in critically ill patients found that statin use in the ICU was associated with reduced delirium in the early stage of sepsis, and later in their ICU stay if the patient was not septic. This study confirms data from a UK cohort analysis of 470 consecutive ICU patients that showed a similar decrease in daily risk of delirium following acute statin administration [28,29] This benefit may be mediated by a reduction of systemic inflammation.

Few small trials support a plausible anti-inflammatory effect of statins used at clinically relevant doses over the short term. One study in 42 patients comparing one dose of 80 mg simvastatin with a placebo, demonstrated significant reductions in serum median and mean CRP concentrations measured 48 hours later [30]. In a trial where a model of acute lung inflammation was induced by inhalation of lipopolysaccharide in 30 healthy human volunteers, simvastatin showed a reduction in systemic inflammation [31] . Another trial compared 17 patients who received standard therapy for unstable angina or non-Q wave myocardial infarction with 13 patients also given one dose of cerivastatin [32]. At 24 hours, the patients treated with statin had significantly lowered CRP levels.

However, there have been no randomized controlled trials to investigate whether simvastatin prevents delirium in critically ill patients. This trial will test the efficacy of simvastatin 80 mg, administered enterally or orally, on reducing delirium in the critically ill. The primary outcome of this study, the number of delirium- and coma-coma free days, is pragmatic and clinically relevant. It addresses the specific issues of mechanically ventilated patients who cannot be assessed for delirium, due to either sedation from a combination of drugs or the patient’s underlying disease, while also accounting for the possibility that a reduction in delirium days may be a result of an increase in coma days, rather than a patient’s brain returning to normal function. In addition, we aim to examine the mechanisms underlying any biological effects of simvastatin in delirium.

For this study we have excluded patients prescribed statins before admission as evidence from a multi-centre randomised trial in patients with severe sepsis did not support stopping statins. Prior statin users randomised to receive placebo had higher 28 day mortality although this was not statistically significant at 90 days. In addition Morandi et al. in their observational study showed that discontinuation of statins in patients was associated with increased delirium.

The dosage of simvastatin administered in this placebo-controlled trial was chosen based on data from a retrospective observational study of statin usage in patients with sepsis, which showed a greater mortality benefit in patients who were receiving a higher dose of statin [33]. A nested cohort study found that statin therapy reduced hospital mortality in patients with sepsis only when higher doses of statins, in particular simvastatin, were given [34]. Moreover, simvastatin at a dose of 80 mg has been shown to be well tolerated in acute lung injury patients, with no increase in adverse events [35].

If the hypothesis that decreasing inflammation with statins will decrease delirium is correct, then patients receiving the statin will show a reduced number of days in delirium compared with the placebo group. Moreover, the inflammatory theory implicated in the pathogenesis of delirium will be strengthened.

## Trial status

The MoDUS trial is ongoing, with 99 patients recruited as of March 2015. Recruitment will continue to November 2016.

## Abbreviations

ALT: Alanine transaminase
β-amyloid: Beta-amyloid
BTACT: Brief Test of Adult Cognition by Telephone
CAM-ICU: Confusion Assessment Method for the Intensive Care Unit
CI: Chief Investigator
CK: Creatine kinase
CONSORT: Consolidated Standards of Reporting Trials
CRF: Case Report Form
CRP: Serum C-reactive protein
CUA: Cost-utility analysis, DMEC, Data Monitoring and Ethics Committee
EQ-5D-5 L: EuroQol-5 dimension-5 level questionnaire
GCP: Good Clinical Practice
ICU: Intensive care unit
IL: interleukin
IQCODE: Questionnaire on Cognitive Decline in the Elderly
MoDUS: Modifying Delirium Using Simvastatin
NHS: National Health Service
NICTU: Northern Ireland Clinical Trials Unit
PerLR: Personal Legal Representative
PRE-DELIRIC: PREdiction of DELIRium in ICu patients
ProfLR: Professional Legal Representative
QALY: quality adjusted life years
RASS: Richmond Agitation and Sedation Score
REC: Research Ethics Committee
SAEs: Serious adverse events
SOFA: Sequential Organ Failure Assessment
SUSARs: Occurrence of suspected unexpected serious adverse reactions
TSC: Trial Steering Committee
